# The Role of Sexually Transmitted Infections in HIV-1 Progression: A Comprehensive Review of the Literature

**DOI:** 10.1155/2013/176459

**Published:** 2013-06-24

**Authors:** Helen M. Chun, Robert J. Carpenter, Grace E. Macalino, Nancy F. Crum-Cianflone

**Affiliations:** ^1^Infectious Disease Clinical Research Program, Uniformed Services University of the Health Sciences, Bethesda, MD, USA; ^2^Naval Health Research Center, San Diego, CA, USA; ^3^Division of Infectious Diseases, Naval Medical Center San Diego, 34800 Bob Wilson Drive, Suite 5, San Diego, CA 92134-1005, USA; ^4^San Diego State University, San Diego, CA, USA

## Abstract

Due to shared routes of infection, HIV-infected persons are frequently coinfected with other sexually transmitted infections (STIs). Studies have demonstrated the bidirectional relationships between HIV and several STIs, including herpes simplex virus-2 (HSV-2), hepatitis B and C viruses, human papilloma virus, syphilis, gonorrhea, chlamydia, and trichomonas. HIV-1 may affect the clinical presentation, treatment outcome, and progression of STIs, such as syphilis, HSV-2, and hepatitis B and C viruses. Likewise, the presence of an STI may increase both genital and plasma HIV-1 RNA levels, enhancing the transmissibility of HIV-1, with important public health implications. Regarding the effect of STIs on HIV-1 progression, the most studied interrelationship has been with HIV-1/HSV-2 coinfection, with recent studies showing that antiherpetic medications slow the time to CD4 <200 cells/µL and antiretroviral therapy among coinfected patients. The impact of other chronic STIs (hepatitis B and C) on HIV-1 progression requires further study, but some studies have shown increased mortality rates. Treatable, nonchronic STIs (i.e., syphilis, gonorrhea, chlamydia, and trichomonas) typically have no or transient impacts on plasma HIV RNA levels that resolve with antimicrobial therapy; no long-term effects on outcomes have been shown. Future studies are advocated to continue investigating the complex interplay between HIV-1 and other STIs.

## 1. Introduction

Individuals infected with human immunodeficiency virus-1 (HIV-1) are often coinfected with other sexually transmitted infections (STIs) due to shared routes of transmission. Over the past decade, there has been mounting evidence of the bidirectional relationship between HIV-1 and other STIs. Initially, studies showed that HIV-1-infected persons may be at risk for more frequent and severe forms of STIs as well as poorer treatment outcomes, especially in cases of concurrent herpes simplex virus-2 (HSV-2) and syphilis infection. More recent data have demonstrated that certain concomitant STIs directly affect HIV-1 transmissibility and may alter HIV-1 control and increase progression to AIDS. This review summarizes the current literature regarding the most common STIs (HSV-2, hepatitis B virus, hepatitis C virus, human papilloma virus, syphilis, gonorrhea, chlamydia, and trichomonas) and their impact on HIV-1 progression.

## 2. Herpes Simplex Virus Type-2

Most persons who are infected with HIV-1 are also infected with HSV-2, with published seropositivity rates of 50–90% [1, 2]. Globally, HSV-2 is the most common cause of genital ulcer disease (GUD), and studies indicate a strong, synergistic relationship between the dual epidemics of HIV-1 and HSV-2 [3]. HSV-2 has been shown to play an important role in the spread of HIV-1 (a 3-fold higher risk of acquisition) [4] and has been estimated to contribute over 25% of incident HIV-1 infections in areas of high HSV-2 prevalence (e.g., Africa) [5]. This increased risk likely occurs through multiple mechanisms, including the presence of mucosal disruption and the influx of cells expressing chemokine receptor 5 (CCR5) [6, 7]. Additionally, there is increased HIV-1 shedding in genital secretions [8] due to local inflammation and the interactions between HSV-2 proteins and the HIV-1 long terminal repeat (LTR) genes and *Tat* protein [9–11]. HSV-2 and HIV-1 can infect the same cells, and HSV-2 proteins ICP-10, ICP-27, and ICP-4 have been shown to upregulate HIV-1 replication by their interactions with the HIV-1 LTR region. Further, HSV-2 protein 16 interacts with the HIV-1 *Tat* protein and increases HIV-1 transcription [9–13]. As a result, HSV-2 may not only enhance HIV-1 transmission, but it may also have a significant impact on HIV-1 viral control and disease progression among coinfected patients.

Regarding the impact of HSV-2 on the HIV-1 coinfected patient, studies have demonstrated that HSV-2 increases both genital and plasma HIV-1 RNA levels. Initial studies demonstrated that HSV-2 reactivation, with the presence of clinical lesions, was associated with transient increases in genital shedding and levels of plasma HIV-1 RNA [14, 25]. Additionally, HSV-2 replication and shedding occur in the absence of symptoms, suggesting that the impact of HSV-2 extends beyond the timing of clinical HSV-2 lesions. Several studies have demonstrated that asymptomatic HSV-2 shedding significantly increases mean genital and plasma HIV-1 RNA levels and results in higher HIV-1 viral load set points [8, 14, 26, 27]. These findings suggest that HSV-2 coinfection may have important implications for both the spread of HIV-1 and HIV-1 virologic control of the coinfected patient.

Given the potential role of HSV-2 on HIV-1 infection, several studies have evaluated the impact of antiherpetic medications on genital and plasma HIV-1 RNA levels in HIV-1/HSV-2 coinfected persons (Table 1) [8, 14–24]. A small study by Schacker et al. (*n* = 12) showed that acyclovir reduced plasma HIV-1 RNA levels among coinfected persons, with an average reduction of 48% [14]. 

Eight randomized studies subsequently examined the impact of antiherpetic suppressive treatment on plasma HIV-1 RNA levels among HIV-1/HSV-2 coinfected persons not receiving combination antiretroviral therapy (CART) (Table 1). The first study found that valacyclovir 500 mg twice daily versus placebo in a cohort of 136 women in Burkina Faso with HIV-1/HSV-2 coinfection reduced both cervical (−0.29 log_10_ copy/mL, 95% confidence interval [CI]: −0.44, −0.15) and plasma (−0.53 log_10_ copy/mL, 95% CI: −0.72, −0.35) HIV-1 RNA levels [8]. Additional studies involving diverse HIV-1 populations are shown in Table 1. In the largest study to date, Celum et al. examined the effect of long-term acyclovir use in a study designed to determine if HIV-1 transmission rates among discordant couples could be reduced with acyclovir [19]. A randomized, placebo-controlled trial of acyclovir (400 mg twice daily for 102 weeks) among 3,408 African heterosexual couples reported a reduction in the mean plasma concentration of HIV-1 by −0.25 log_10_ copies/mL (95% CI: −0.29, −0.22; *P* < 0.001) in men and women, although the study failed to show a reduction in HIV-1 transmission rates. Mugwanya et al. evaluated whether higher doses of antiherpetic medications were more effective at reducing HIV-1 RNA levels [21]. A randomized, crossover trial of two types and doses of antiherpetic medications was evaluated (valacyclovir 1.5 g versus acyclovir 400 mg twice daily for 12 weeks) in 32 HIV-1/HSV-2 dually infected Kenyan individuals with CD4 cell counts >250 cells/mL and not on CART. Mean plasma HIV-1 levels were significantly lower in the valacyclovir compared with the acyclovir arm: −0.62 log_10_ copies/mL (95% CI: −0.68, −0.55; *P* < 0.001, a 76% decrease), and valacyclovir decreased the viral load by >1 log compared with baseline pretreatment values. This study suggested that higher doses of antiherpetic medications may offer greater benefit in reducing HIV-1 RNA levels and revealed no increase in adverse events using the higher dose.

In summary, these studies examined both male and female HIV-1/HSV-2 coinfected patients from a variety of geographic locations and demonstrated that antiherpetic medications (typically using 400–800 mg of acyclovir twice daily or valacyclovir 500 mg twice daily) for 1–3 months reduced the plasma HIV-1 levels by 0.26 to 0.47 log_10_ copies/mL among HIV-1 patients not receiving CART.

A meta-analysis recently summarized the randomized evidence (2000–2009) regarding the association between antiherpetic medications and plasma HIV-1 RNA levels [28], with a summary effect estimate of −0.33 (95% CI: −0.56, −0.10) log_10_ copies, an approximate halving of HIV-1 plasma viral load. Of note, characteristics associated with a larger decrease in HIV-1 viral load included older median age, valacyclovir use, and higher compliance rates [28]. These randomized studies clearly show the benefit of antiherpetic medications in reducing HIV RNA levels in the setting of asymptomatic HSV-2 coinfection. The reduction in HIV-1 RNA loads by these agents is due to suppression of HSV-2 replication and its interaction with the HIV-1 virus, as described above, as well as the potential direct antiretroviral effects of acyclovir [29–31].

Unlike prior studies that examined the impact of antiherpetic therapy among HIV-1 patients not receiving CART, a randomized, double-blind, and placebo-controlled trial of valacyclovir 500 mg twice a day in HIV-1/HSV-2-infected women on CART, with a similar design as the study by Nagot et al. [8], showed no significant impact on the frequency or quantity of genital HIV-1 RNA [22]. The plasma HIV-1 RNA was reduced (−0.41 log_10_ copy/mL), although this did not reach statistical significance, perhaps due to small sample size. Further studies are needed to examine the benefit of antiherpetic medication on systemic HIV-1 control in this setting.

While these studies demonstrated that antiherpetic therapy reduces genital and plasma HIV-1 RNA levels among patients not receiving CART [8, 14–21], whether this therapy could slow HIV-1 clinical progression was not specifically examined. Since higher plasma HIV-1 RNA levels are associated with faster HIV-1 disease progression [32], it was hypothesized that HSV-2 suppression may slow HIV-1 progression. A recent review noted that a reduction in HIV-1 viral load of 0.3 log_10_ or 0.5 log_10_ may slow HIV-1 clinical progression by 25% to 44%, respectively [33]. Additionally, during early trials of antiretroviral therapy (ART), acyclovir was associated with increased survival [34]. Hence, clinical trials were undertaken to determine if the use of acyclovir could slow HIV-1 progression.

Lingappa et al. [23] randomized 3,381 HIV-1/HSV-2 dually infected heterosexuals to acyclovir 400 mg twice daily or placebo for 24 months (Table 1). All participants had a CD4 cell count ≥250 cells/*μ*L and were not receiving CART. HIV-1 progression was defined as a CD4 cell count <200 cells/*μ*L, CART initiation, or a nontrauma-related death. The receipt of acyclovir was associated with a 16% reduction in HIV-1 progression (hazard ratio [HR] = 0.84, 95% CI: 0.71, 0.98, and *P* = 0.03), as well as with a delayed risk of reaching a CD4 cell count <350 cells/*μ*L (HR = 0.81, 95% CI: 0.71, 0.93, and *P* = 0.002). Assuming that the incidence of endpoints remained constant over time, acyclovir would have delayed the median time to an endpoint by estimated 10.7 months. A second study on HIV-1 progression [24] investigated the effect of daily suppressive acyclovir on HIV-1 disease progression in patients with CD4 cell counts of 300–400 cells/*μ*L who were not receiving CART. Participants were randomized to acyclovir 400 mg twice daily or placebo and followed for 24 months. The treatment group had a 25% reduced risk of developing a CD4 cell count <250 cells/*μ*L or initiating of CART for WHO stage 4 disease. In addition to these studies [23, 24], one study evaluated the impact of acyclovir (400 mg twice daily) versus placebo on the viral load set point during early HIV-1 infection (*n* = 76) but found no significant effect [35].

Overall, these studies provide strong evidence that antiherpetic medications reduce both plasma and genital HIV-1 RNA levels among chronically infected HIV-1 patients who are coinfected with HSV-2. Antiherpetic medication may offer an important and viable strategy to reduce HIV-1 progression in the setting of limited CART availability or among patients wishing to remain off CART. Advantages of antiherpetic medication use in these settings include its low cost (i.e., available as a generic medication), excellent tolerability, lack of need for specific laboratory monitoring, and low frequency of adverse events. Therapy is most advantageous when utilized continuously, since the interruption of therapy is associated with viral rebound [21]. The effect of antiherpetic medication on plasma HIV-1 concentrations can be seen within a week of initiation [21], with no reduction in efficacy noted over time [23]. The added value of antiherpetic medications concurrently with CART or in other clinical scenarios (at time of seroconversion or among HIV-2 patients) requires further evaluation. 

## 3. Hepatitis B Virus

Hepatitis B virus (HBV) is more common in HIV-1-infected individuals than in the general population due to shared risk factors for acquisition [36–39], with published rates of 6–14% having concurrent chronic HBV [40]. Current evidence suggests that HIV-1 infection modifies the course of HBV with an adverse impact on HBV-related liver disease progression, including higher serum HBV DNA polymerase activity; lower rates of loss of serum hepatitis B e antigen (HBeAg); and increased risk of cirrhosis, liver-related mortality, and hepatocellular carcinoma, especially among patients with lower CD4 cell counts [36, 41–46]. HBV infection is also more likely to become chronic in those coinfected with HIV-1 [43].

Overall, studies evaluating the influence of HBV/HIV coinfection on HIV RNA suppression, immunologic CD4 cell count recovery, and clinical outcomes in individuals on HAART have been limited and conflicting, with several studies finding no substantial impact of HBV coinfection on immunologic or HIV virologic responses to ART [46–53]. Other studies, however, have shown a significant impact on ART outcomes [54–56]. Conflicting findings from these studies may be attributable to the inconsistent choice of viral markers for study eligibility [46, 55], being observational versus prospective studies, having limited follow-up time [46, 55], small numbers of HIV/HBV coinfected patients, limited mortality data [57], varying HBV disease characteristics (HBeAg status, HBV DNA), varying endemicity of HBV, HCV coinfection, lack of data on opportunistic infections [53], and lack of data to specify if patients were receiving HBV-active ART. Clinical studies prior to the general availability of CART evaluating the impact of HBV on HIV-1 progression have also shown inconsistent results [48, 49, 51, 54, 56, 58–60]. Some studies found no differences in HIV-1 progression between those with and without chronic HBV [36, 53, 59, 61], while other studies have shown that chronic HBV may negatively impact HIV-1 progression with a significant increased risk of AIDS or death [55, 62–64]. HBV is thought to mediate destruction of CD4 cells through T-cell activation or splenic sequestration seen in advanced liver disease [53].

Studies on CD4 cell counts at and after ART initiation are conflicting. Some have shown that HIV-1/HBV coinfected patients have significantly lower CD4 cell counts at ART initiation compared with individuals infected with HIV-1 alone [46, 48, 53, 55, 65, 66]. In a study from China, HBeAg positivity, rather than HBV DNA level, was associated with lower cell counts in chronic HIV-1/HBV coinfected patients [65]. Other studies have not detected differences in CD4 cell counts prior to ART initiation [49, 54, 67]. In one study, individuals with occult hepatitis B (HBV DNA present in the absence of hepatitis B surface antigen) demonstrated lower CD4 cell counts as compared to individuals without occult hepatitis B [68].

Some studies evaluating CD4 cell count responses in coinfected individuals after ART found no differences in CD4 gains in HIV-1/HBV coinfected versus HIV-1 monoinfected individuals [54, 57, 69], while other studies have shown a negative impact of HBV on CD4 cell count recovery. However, the studies by Law and Hawkins showed no difference by 48 weeks and borderline significance at 12 months, respectively [46, 55, 56, 67]. Individuals with HBeAg-positive status at ART initiation and HBV DNA ≥20,000 IU/mL were significantly associated with lower CD4 cell counts, but HBV status and HBV DNA level were not shown to affect CD4 cell count rise [53]. One hypothesis why lower CD4 cell counts may be observed with HBeAg and higher HBV DNA is the possibility that HBV replication increases HIV-1 replication, in turn lowering CD4 cell counts based on in vitro data showing HBV X protein acting as a transactivator of HIV-1 transcription, but these data have not been demonstrated in vivo [70–72]. An alternative explanation is that HBV leads to increased apoptosis of CD4 cells through increased T-cell activation or an alternation in cytokines that leads to decreased production or destruction of CD4+ T cells [53].

 In addition to the potential effects on CD4 cell counts, several studies have examined the impact of coinfection on the plasma HIV-1 viral load. Evaluation of HIV-1 viral load at ART initiation has not shown any differences, but there are conflicting data on virologic suppression, with some studies showing no differences in the proportion of individuals achieving virologic suppression over time [55, 57, 65–67]. For example, a lower proportion of HBeAg-positive individuals achieved VS (HIV-1 VL ≤400 copies/mL) at 24 weeks compared with HBeAg-negative or HIV-1 monoinfected individuals, but no differences were observed by 48 weeks [53]. Additionally, in this study from Nigeria, the proportion of patients with virologic suppression (HIV-1 VL ≤400 copies/mL) at 6 months was 67% versus 70% in the HIV-1/HBV coinfected and HIV-1 monoinfected groups, respectively [53]. Finally, one study showed a higher rate of virologic failure in HBV/HIV-1 coinfected patients, although the cause for the higher rate was not clear [54]. 

 There have been no reported differences in the incidence of new AIDS-defining events among HBV/HIV-1 coinfected compared with HIV-1 monoinfected individuals [46, 54, 73]. Nevertheless, all-cause mortality has been shown to be higher, most commonly attributable to liver-related deaths [42, 46, 73]. A meta-analysis (including 11 studies with 12,382 patients) showed a significantly increased risk for all-cause mortality in coinfected patients [73]. Studies evaluating the impact of HBV-active ART on mortality are limited. One study conducted in an urban Tanzanian cohort showed a significantly higher risk of mortality (HR = 1.28, 95% CI: 1.02–1.61, and *P* < 0.03) in coinfected patients compared with monoinfected HIV-1 patients on ART regimens that did not contain tenofovir (TDF), but no difference in mortality among the two groups with the use of TDF-containing regimens [55].

Given the findings from a recent multinational cohort showing HIV-1/HBV coinfected individuals have significantly lower CD4 cell counts than monoinfected patients, determining the HBV status at HIV diagnosis and prior to CART initiation should be a priority [66]. Despite the dramatic reductions in morbidity and mortality in the CART era, lower survival rates in HBV/HIV-1 coinfected individuals are seen, with death attributable to chronic hepatic complications assuming more prominence in the era of CART [54]. Given the differences in findings from the studies described above, further evaluations of the long-term immunologic and virologic responses to ART in HBV/HIV-1 coinfected individuals compared with HIV-1 monoinfected individuals are needed to further our understanding of the effect of HBV on HIV-1 and ART response and long-term outcomes.

## 4. Hepatitis C Virus

Hepatitis C virus (HCV) is a common blood-borne pathogen among HIV-1-positive intravenous drug users, with recent increasing rates via sexual transmission among men who have sex with men (MSM) [74–76]. The negative impact of HIV-1 infection on the natural history of HCV infection is well established; HCV-associated liver disease, including fibrosis, cirrhosis, and end-stage liver disease, is accelerated among HIV-1-infected populations.**  **For example, progression to cirrhosis is 2-3 times higher in coinfected than monoinfected individuals, with a third of coninfected patients estimated to progress to cirrhosis in less than 20 years [77]. An increased risk of liver-related deaths among coinfected compared with HCV monoinfected drug users despite CART use has also been reported from a recent 20-year prospective study [78].

While the mechanism to explain the adverse impact of HIV-1 on liver disease in HCV-infected individuals is not clear to date, it likely includes immune activation, apoptosis, and diminished HCV-specific T-cell responses [79–81]. Increased tissue damage in coinfected populations may be due to the accumulation of cytotoxic CD8 cells in the liver that may increase inflammatory mediators [82, 83]. HIV-1 replication has been noted in hepatocytes and hepatic stellate cells [81, 84, 85], with promotion of collagen expression and secretion of proinflammatory cytokines [85]. Insulin resistance appears to be critical in liver steatosis and liver disease progression, although the data are not definitive if it is more prevalent among coinfected [86] or monoinfected individuals [87]. 

The debate as to whether HCV has a negative impact on HIV-1 disease progression continues. While many studies have shown higher mortality among HIV-1/HCV coinfected individuals compared with those with mono-infection, a meta-analysis conducted in 2009 that included 100,000 patients across 30 studies found no increase in mortality in coinfected patients prior to the CART era. After CART, this study found an increased risk for overall mortality but not for AIDS-defining conditions [88]. However, some studies have found an increased risk of AIDS and AIDS-related infections; data from a cohort in Italy reported a twofold increase in AIDS risk among coinfected participants [89], with increases in bacterial and mycotic infections. Similarly, a US cohort of HIV-1-positive women also found an almost twofold increase in risk of AIDS for those never on CART [90].

Coinfected patients have been found to have high levels of T-cell activation even following CART compared with monoinfected patients [90–92]. Chronic immune activation may cause immune dysfunction and cytokine production, leading to enhanced HIV-1 and HCV replication and lower CD4 cell counts [91]. In one study of HIV-1-infected women, HCV-viremic compared with HCV-uninfected women had high levels of activated CD8 T cells associated with incident AIDS, and AIDS in both groups was associated with CD4 activation [90, 91], while suppression of HCV with therapy reduces activation [92]. HCV coinfection may increase immune activation, leading to CD4 cell apoptosis in HIV-1-untreated patients and more rapid progression to severe immunodeficiency [93]. However, the impact of HCV infection on CD4 cell recovery following CART is conflicting; some reports note a poorer CD4 response in coinfected compared with monoinfected patients [94], while others do not [93–98].

It is important to consider the timing of CART initiation relative to anti-HCV therapy for coinfected patients. CART may slow liver disease progression and might therefore be initiated earlier in coinfected than in HIV-1 monoinfected patients [99, 100]. Conversely, CART may increase fibrosis in coinfected patients through cumulative hepatotoxicity [99, 101]. Recent guidelines recommend that CART generally be initiated first to slow liver disease progression and increase CD4 cell count, bearing in mind that some drugs should be avoided while others should be monitored for hepatotoxicity [102]. 

## 5. Human Papilloma Virus

Human papilloma virus (HPV) is known to be the causative agent for urogenital warts, oropharyngeal cancer, cervical dysplasia and cancer in women, and anal dysplasia and cancer in men and women [103–106]. Cervical and anal cancers develop from cervical intraepithelial neoplasia (CIN) and anal intraepithelial neoplasia (AIN), respectively. Most studies in the medical literature demonstrate adverse effects of HIV-1 infection on HPV disease and its progression. In fact, HIV-1-infected persons tend to have larger and multicentric venereal warts, have more anal and cervical dysplasia, and tend to be infected with multiple HPV subtypes [107–110]. 

HIV-1-infected persons are also at higher risk for intraepithelial neoplasia and cancers. Women with HIV-1 infection are approximately 30 times more likely to develop CIN (compared with HIV-1-uninfected women) [107], and MSM with HIV-1 infection are approximately 6–9 times more likely to develop AIN (compared to HIV-1-negative MSM) [111]. Of note, anal sex is not a prerequisite for AIN. Conley et al. found the following AIN prevalence among their HIV-1-infected cohort: 9–31% in MSM, 3–17% in women, and 1–9% in men who have sex with women [112], while another study of HIV-1-infected women found an AIN prevalence of 33% over 3 years of follow-up [113]. HIV-1-infected persons are also at higher risk of developing anal cancer compared with their HIV-1-uninfected counterparts. Crum-Cianflone et al. found anal cancer incidence rate of 128/100,000 among a large cohort of HIV-1-infected persons compared with 1.4/100,000 for men in the general US population [114]. 

 There are limited data implicating HPV infection as a risk factor for acquiring HIV-1 infection [115, 116] with a systematic review in 2012 (examining six studies in women and two in men) showing a twofold increased risk of HIV-1 acquisition in women, with similar trends in the men [115]. However, regarding the potential direct effect of HPV on HIV-1 infection control (e.g., HIV RNA levels) or noncancer-related clinical progression (e.g., time to AIDS), our review did not identify any published studies to date. 

## 6. Syphilis

Syphilis represents the second most common cause of GUD among HIV-1-infected persons, with documented increasing rates of syphilis among HIV-1-infected persons over the past decade, especially among MSM [117–119]. Multiple studies have confirmed that syphilis infection is associated with an increased risk of acquiring and transmitting HIV-1 [120, 121], with mechanisms including the disruption of the mucosa and the influx of CCR5+ cells. Regarding the impact of HIV-1 infection on syphilis, studies have shown that it adversely affects the serologic response to syphilis treatment, especially at lower CD4 cell counts <200 cells/*μ*L, but that CART reduces the failure rate [122, 123].

 Several studies have evaluated the impact of syphilis on the natural history of HIV-1 infection. Initial studies focused on the effect of syphilis coinfection on CD4 cell counts and HIV-1 RNA plasma levels. The first published report demonstrated small changes in HIV-1 RNA levels and CD4 counts associated with syphilis infection; Buchacz et al. showed that among 52 HIV-1-infected men in California with primary or secondary syphilis, within-person changes in viral loads were slightly higher during versus prior to the syphilis infections (mean 0.22 log_10_ copies/mL, *P* = 0.02) that decreased after therapy (−0.10 log_10_ copies/mL, *P* = 0.52) [124]. A small decrease in CD4 cell counts was also noted during infection (−62 cells/*μ*L, *P* = 0.04), with recovery after treatment (+33 cells/*μ*L, *P* = 0.23).

 A second study was conducted in Denmark among 41 HIV-1-infected persons with primary or secondary syphilis. This study found an increase in HIV-1 RNA levels and a reduction in CD4 cell counts among those with an initial CD4 count of >500 cells/*μ*L [125], which improved after the treatment of syphilis. Additionally, a study from Spain demonstrated that, among 118 coinfected patients, syphilis resulted in similar CD4 cell and viral load effects among one-third of patients [126]. Specifically, among those with a detectable viral load before syphilis, infection resulted in an increase in HIV-1 viral load of 1.03 log_10_ (IQR 0.64–1.32), and 25% of those initially suppressed had viral load detection during syphilis infection. Mean CD4 counts were lower during syphilis than before (496 versus 590 cells/*μ*L, *P* < 0.001) and increased after treatment (597 versus 509 cells/*μ*L, *P* < 0.001) [126]. Of note, >50% of patients in each of these studies [124–126] were on ART. Although most studies have shown that syphilis adversely impacts HIV-1 control, some studies have shown no effect, including a study of 38 coinfected persons from Italy [127]. Further, a study examining 63 coinfected persons and a group of controls (without an STI) from London showed no major impact on HIV-1 RNA levels in the blood or semen, but it did show changes in CD4 cell counts among early latent syphilis cases [128].

The most recent study examined a cohort of HIV-1 patients 1998–2006 and compared 282 coinfected patients with 1,233 syphilis-free matched HIV-1 controls [129]. This study showed that plasma HIV-1 RNA increases (adjusted odds ratio = 1.87, 95% CI: 1.40–2.49) and CD4 cell decreases (−28 cells/*μ*L, *P* = 0.001) were more likely among those with syphilis infection. Further, this study demonstrated the association between syphilis and viral rebound among patients who were receiving effective CART regimens. These findings were independent of the syphilis stage or initial CD4 cell count. The potential mechanism of these effects has been hypothesized to be a result of immune activation of host cells, increase in cytokine secretion, and upregulation of chemokine coreceptors [126, 130].

In order to evaluate the potential effect of syphilis on HIV-1 progression (time to AIDS or death), a study by Weintrob et al. prospectively evaluated 2,239 HIV-1 seroconverters not receiving ART with confirmed (9.2%) and probable (2.9%) syphilis [131]. This study, with 7,827 person-years of follow-up, however, found no impact of syphilis on HIV-1 disease progression (HR = 0.99, 95% CI: 0.73–1.33). Overall, syphilis increases the risk of HIV-1 transmission and leads to transient increases in HIV-1 RNA plasma levels and decreases in CD4 counts among a subset of patients regardless of the receipt of ART. Treatment of syphilis leads to resolution of the viral load and CD4 cell changes with no apparent long-term impact on HIV-1 progression.

## 7. Gonorrhea and Chlamydia Infections

*Neisseria gonorrhoeae* (GC) and *Chlamydia trachomatis* (CT) are bacteria that may be sexually transmitted from person to person and cause pharyngitis, cervicitis/urethritis, epididymitis, proctitis, and pelvic inflammatory disease. Both men (including MSM) and women often harbor asymptomatic infection [132]. The interrelationships of these bacteria and HIV-1 are complex, but exacerbation of either infection and facilitation of HIV-1 transmission have been demonstrated at some, but not all, mucosal surfaces. HIV-1-infected persons may have reduced clearance of these infections at mucosal sites. For example, interferon gamma (IFN-*γ*) is thought to be important for the clearance of CT infection [133–135]; however, studies suggest that HIV-1-infected persons secrete significantly less (IFN-*γ*) [136], suggesting impaired elimination of these infections.

Concurrent bacterial STIs have been shown to increase HIV-1 shedding at mucosal sites, with subsequent treatment resulting in a decrease in the amount of HIV-1 present in genital fluids [137–140]. A study comparing seminal HIV-1 levels among HIV-1 patients (not on CART) with GC/CT, nonspecific urethritis, or no STI found that the presence of GC or CT resulted in a fivefold increase in seminal HIV-1 RNA levels that were not observed in the other groups [138]. Following antibiotic therapy, HIV-1 RNA levels decreased in the GC/CT group. In a second study, the effects of GC/CT were evaluated among an HIV-1 cohort on ART, finding that most patients with undetectable plasma HIV-1 viral loads maintained no detection of HIV-1 in seminal fluid after acquiring GC/CT [139]. Similarly, a study of MSM receiving CART who had a bacterial STI showed that the plasma HIV-1 RNA level was the only significant correlate of rectal viral load in a model that included concurrent GC and CT infection [141]. However, presence of GC or CT infection strengthened the correlation between plasma and rectal viral load; thus these coinfections may enhance rectal shedding in setting ongoing viral replication. These data suggest that the impact of GC/CT urethritis on changes in genital HIV-1 RNA levels is limited in the setting of effective ART, but substantial in patients not receiving CART and who have detectable plasma HIV-1 viral loads, potentially enhancing HIV-1 infectivity.

Regarding the effect of GC/CT on HIV-1 plasma viral load, Nkengasong et al. studied HIV-1-infected female sex workers in Africa and found that STIs, specifically ulcerative disease and GC, caused a 2.5-fold rise in HIV-1 plasma viral load [142]. They also found that those with STIs tended to demonstrate more CD4 cell activation and increased proinflammatory cytokines, but they did not reach statistical significance. Anzala et al. also studied female sex workers in Africa [143] and found that acute infection with GC caused transient increases ininterleukin- (IL-) 4, IL-6, IL-10, soluble tumor necrosis factor- (TNF-) *α*, and HIV-1 plasma viral load and a decline in CD4 cell counts, which returned to baseline after the acute bacterial infection was treated. They also observed similar changes in women with acute pelvic inflammatory disease. 

These data suggest that coinfection with bacterial STIs may acutely impact HIV-1 control and increase transmissibility of the virus. To date, there are no data to suggest that GC/CT infections impact the long-term progression of HIV-1 disease.

## 8. Trichomoniasis

 Trichomoniasis, caused by the protozoan parasite,* Trichomonas vaginalis,* is the most common curable, nonviral STI worldwide [144–147], with over 170 million cases per year [148]. *T. vaginalis* is a highly prevalent STI among HIV-1-infected patients [149, 150], and there is a high frequency of asymptomatic or subclinical infection [151–153]. *T. vaginalis* infections are not currently reported to state agencies in the United States, limiting available prevalence data. The advent of polymerase chain reaction (PCR) testing, as a much more sensitive diagnostic technique, has allowed for a greater understanding of the global epidemiology of *T. vaginalis *and has heightened the awareness of the potential impact trichomoniasis has on HIV-1 transmission and female reproductive health [145]. 

 Data have demonstrated that *T. vaginalis *infection enhances HIV-1 transmission [151, 154–156], with risk increased up to threefold [148, 154, 155, 157–159]. One recent study using mathematical modeling found 23% of HIV-1 transmission events from HIV-1-infected women may be attributable to *T. vaginalis* infection [160]. Proposed mechanisms by which *T. vaginalis* infection may increase HIV-1 infection include inducing the inflammatory response of vaginal, exocervix, and urethral epithelia; disrupting mucosal barrier function; recruitment of CD4 lymphocytes and macrophages; development of microhemorrhages; degrading secretory leukocyte protease inhibitors; and enhancing susceptibility to bacterial vaginosis or other abnormal vaginal flora that may increase the risk of HIV-1 acquisition [161–164].

The hemorrhages and inflammation produced by *T. vaginalis* infection in an HIV-1-infected individual can increase the level of virus-laden body fluids and/or the numbers of HIV-1-infected macrophages and lymphocytes in genital areas, thereby amplifying the probability of HIV-1 exposure and transmission [161]. Increased cervical shedding of HIV-1 has been shown to be associated with cervical inflammation [165, 166]. Similarly, studies have shown that men with urethritis have higher HIV-1 RNA concentrations in semen if infected with *T. vaginalis *than those with urethritis of an unidentified cause [167]. Data regarding HIV-1 patients receiving ART have shown that detection of vaginal HIV-1 RNA was not different before or during a *T. vaginalis* infection, suggesting that CART generally maintains low or undetectable genital HIV-1 levels, even in the presence of this STI [168].

 Studies have shown that treatment for trichomoniasis significantly reduces HIV-1 RNA genital shedding [169–171]. These data have important implications for the importance of screening and early treatment of trichomoniasis to decrease viral shedding and possibly decrease HIV-1 transmission risk [170]. 

 The impact of *T. vaginalis* infection on HIV-1 outcomes (immunologic, virologic, and clinical [AIDS or death]) is less well defined. Given the impact of both HIV-1 and *T. vaginalis* on immune activation locally and systemically, it can be hypothesized that coinfection alters immune responses and may alter either CD4 cell count or HIV-1 viral load, but there are no data to support this. Similarly, the coinfection's interaction with the immune system and the enhanced or altered susceptibility to other infections that impact HIV-1 outcomes are plausible.

 Despite the limited data on the impact of *T. vaginalis *infection on HIV-1 progression, it is clear that *T. vaginalis *has a substantial impact on the spread of HIV-1. The potentially large reservoir of asymptomatic carriers, the availability of treatment for trichomoniasis, and the overlap of HIV-1 epidemics throughout communities and the world should prompt policy makers to consider screening and treatment programs for *T. vaginalis* and prompt greater research in understanding the impact trichomoniasis has on HIV-1 outcomes. 

## 9. Summary

Complex bidirectional relationships exist between HIV-1 and other STIs. While the impact of HIV-1 infection on the clinical presentations and treatment outcomes of STIs is well known, fewer data exist regarding the impact of concurrent STIs on HIV-1 progression (virologic, immunologic, and clinical) and acquisition, with most studies focusing on HIV-1 shedding at genital mucosal sites. Studies have shown that the presence of some STIs, both ulcerative and nonulcerative, increases genital HIV-1 RNA levels and enhances the transmissibility of HIV-1, with important public health implications. Studies have also shown that these effects are substantially limited among HIV-1 patients receiving effective ART, suggesting an important role for early ART initiation. Additionally, safe-sex counseling, routine STI screening, and early treatment are critically important.

Regarding the effect of STIs on HIV-1 progression, the most studied interrelationship has been HIV-1/HSV-2 coinfection. Studies have shown that HSV-2 increases genital and plasma HIV-1 RNA levels and that the use of antiherpetic medications reduces these effects and reduces HIV-1 progression among coinfected patients not receiving CART. The impact of other STIs on HIV-1 progression is less clear, but those coinfected with HBV or HCV appear to have higher mortality rates (predominantly HBV/HCV related), during the CART era, but not necessarily higher rates of AIDS progression. For other treatable, nonchronic STIs (i.e., syphilis, gonorrhea, and chlamydia), their impact on HIV-1 RNA levels and CD4 cell counts are typically transient and resolve with antimicrobial therapy. Future studies are needed to continue to define the rates of coinfections in various HIV-1 populations, the pathogenesis and impact of STIs on HIV-1 outcomes, and the role of STI therapy on reducing HIV-1 progression.

## Figures and Tables

**Table 1:** Studies evaluating the impact of HSV-2 suppressive treatment on HIV-1 plasma RNA levels and progression.

First author and reference	Publication year	Sample size	Study	Location	Intervention	Genital HIV-1 RNA change (log_10_ copy/mL)	Plasma HIV-1 RNA change (log_10_ copy/mL)	Clinical outcomes
Studies examining the impact on plasma and/or genital viral load coinfected patients off CART								
Schacker [14]	2002	12 (no CART); 1 patient on dual therapy	Single-arm study	USA	Acyclovir 800 mg three times daily for 8 weeks; no treatment during the preceding and subsequent 8-week periods	NR	48% reduction in RNA levels	NR

Nagot [8]	2007	136 women (no CART)	Randomized study	Burkina Faso	Valacyclovir 500 mg twice daily versus placebo for 12 weeks	Cervical: −0.53 (95% CI: −0.72, −0.35)	−0.29 (95% CI: −0.44, −0.15)	NR

Zuckerman [15]	2007	20 MSM (no CART)	Placebo-controlled crossover	Lima, Peru	Valacyclovir 500 mg twice daily versus placebo (8 weeks for each arm)	Rectal levels: −0.16 (95% CI: −0.07, −0.25; *P* = 0.0008), 33% decrease	−0.33 (95% CI: −0.23, −0.42; *P* < 0.0001), 53% decrease	NR

Dunne [16]	2008	67 women (no CART)	Placebo-controlled crossover	Chiang Rai, Thailand	Acyclovir 800 mg twice daily versus placebo (1 month each phase)	Cervicovaginal levels: −0.3, *P* < 0.01	−0.47, *P* < 0.01	NR

Baeten [17]	2008	20 women (no CART)	Placebo-controlled crossover	Peru	Valacyclovir 500 mg twice daily versus placebo (8-week treatment)	Cervical levels: −0.35, a 55% decrease, and *P* < 0.001	−0.26, a 45% decrease [*P* < 0.001]	NR

Delany [18]	2009	300 women (no CART)	Randomized study	South Africa	Acyclovir 400 mg twice daily versus placebo (3 months)	Cervicovaginal levels: 0.13 (95% CI: −0.14, 0.39)	−0.34 (95% CI: −0.15, −0.54)	NR

Celum∗ [19]	2010	3,360 heterosexual men and women (no CART)	Randomized study	South and East Africa	Acyclovir 400 mg twice daily for up to 102 weeks	NR	−0.25 (95% CI: −0.29, −0.22), *P* < 0.001	NR

Tanton [20]	2010	484 women (no CART)	Randomized study	Tanzania	Acyclovir 400 mg twice daily versus placebo for 6 months	Cervicovaginal levels: −0.01 (95% CI: −0.20, 0.19)	0.04 (95% CI: −0.07, 0.15)	NR

Mugwanya [21]	2011	32 men and women (no CART)	Placebo-controlled crossover	Kenya	Valacyclovir 1.5 g twice daily versus acyclovir 400 twice daily (12 weeks)	NR	−0.62 (95% CI: −0.68, −0.55, *P* < 0.001)	NR

Studies examining the impact on plasma and/or genital viral load coinfected patients on CART								
Ouedraogo [22]	2006	60 women (on CART)	Randomized study	Burkina Faso	Valacyclovir 500 mg twice daily versus placebo for 12 weeks	Cervicovaginal levels: −0.41 (95% CI: −1.35, 0.53)	−0.33 (95% CI: −0.81, 0.16)	NR

Studies examining the impact on clinical HIV-1 progression								
Lingappa∗ [23]	2010	3,381 heterosexual men and women (no CART)	Randomized study	East and Southern Africa	Acyclovir 400 mg twice daily versus placebo for 24 months	NR	∗	16% reduction in HIV-1 progression (HR 0.84, 95% CI: 0.71, 0.98)

Reynolds [24]	2012	440 (no CART)	Randomized study	Uganda	Acyclovir 400 mg twice daily versus placebo for 24 months	NR	NR	25% reduction in progression (HR: 0.75, 95% CI: 0.59, 0.99)

^*^Utilized same study population.

CART: combination antiretroviral therapy; CI: confidence interval; HR: hazard ratio; NR: not reported.
